# Supplementary material for: Study on Permeability Characteristics of Porous Transparent Gels Based on Synthetic Materials
Source: Polymers (Basel). 2021 Nov 19;13(22):4009. doi: 10.3390/polym13224009 (PMC8625236; doi:10.3390/polym13224009)
Supplement: Supplementary file 1 [file polymers-13-04009-s001.zip › polymers-1420797--supplementary.pdf]

Supplementary materials

1. Laponite RD powders physical photos

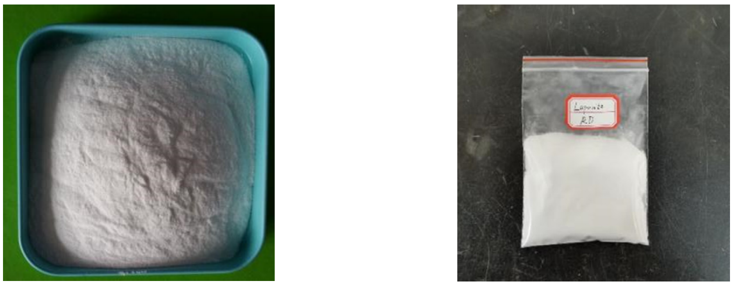

Fig. S1. Laponite RD powders

2. Transparent clay specimen preparation

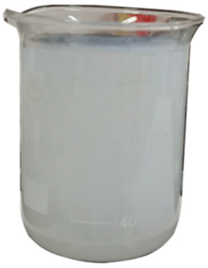

(a)white suspension

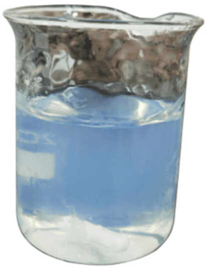

(b)Gel-like substance

Fig. S2. Transparent clay preparation

3. Permeability coefficients of specimens at different permeability pressure differences

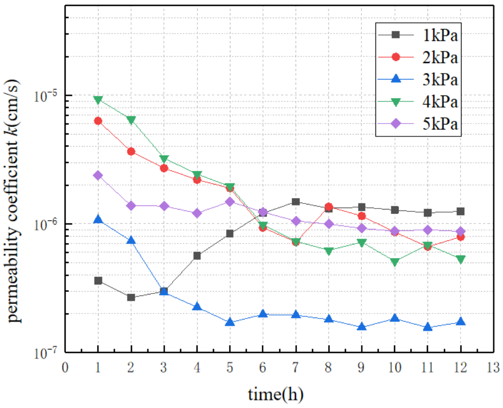

(a)

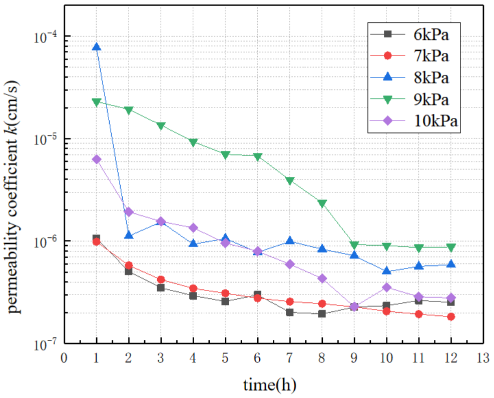

(b)

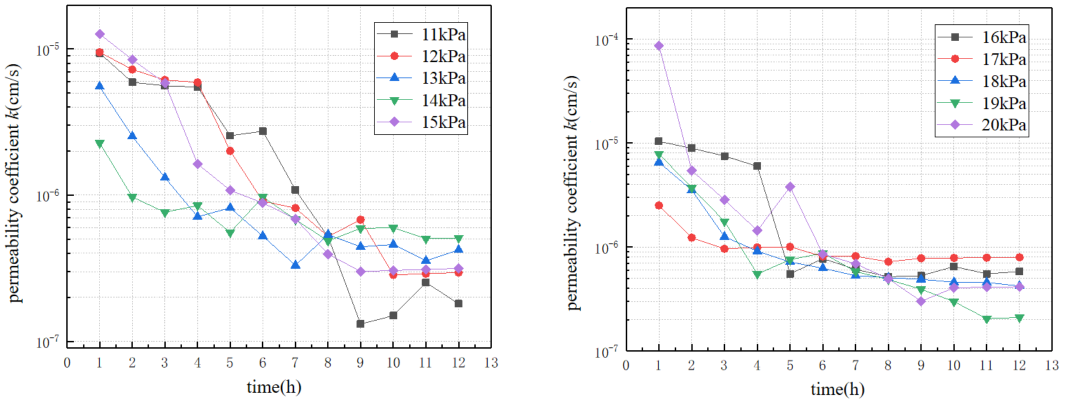

(c) (d)

Fig. S3. The change of permeability coefficient of group A specimens under different pressure difference:(a)1-5kPa; (b)6-10kPa; (c)11-15kPa; (d)16-20kPa.

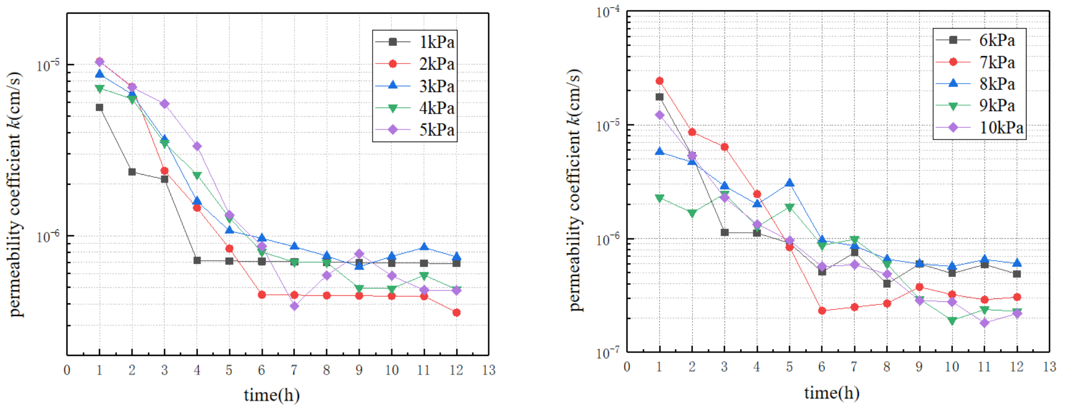

(a) (b)

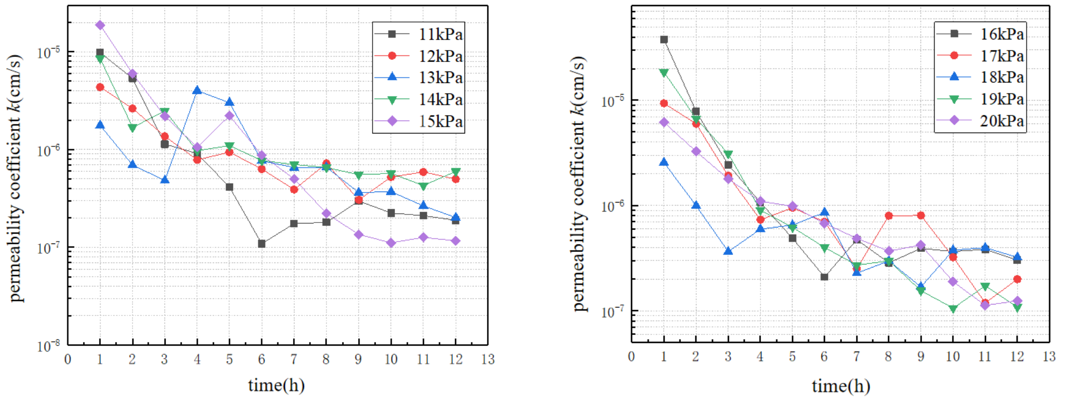

(c) (d)

Fig. S4. The change of permeability coefficient of group B specimens under different pressure differences: (a)1-5kPa; (b)6-10kPa; (c)11-15kPa; (d)16-20kPa.

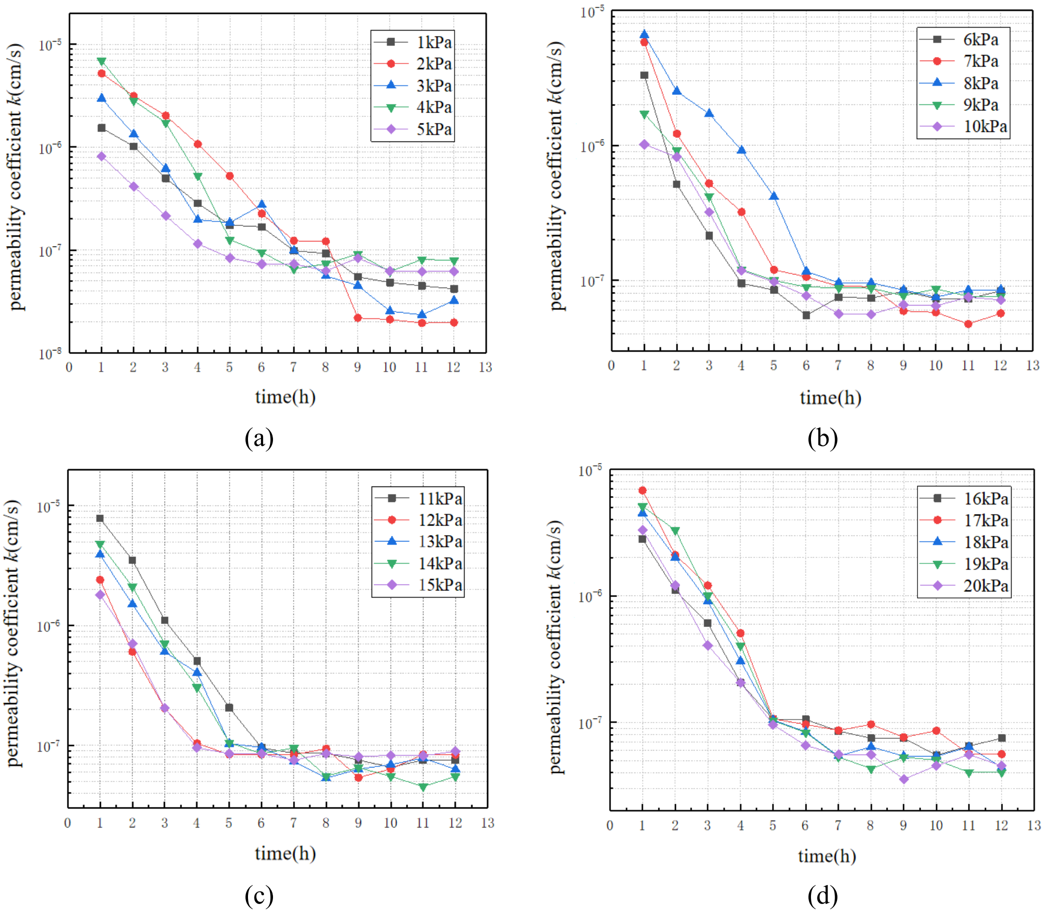

Fig. S5. The change of permeability coefficient of group C specimens under different pressure differences: (a)1-5kPa; (b)6-10kPa; (c)11-15kPa; (d)16-20kPa.

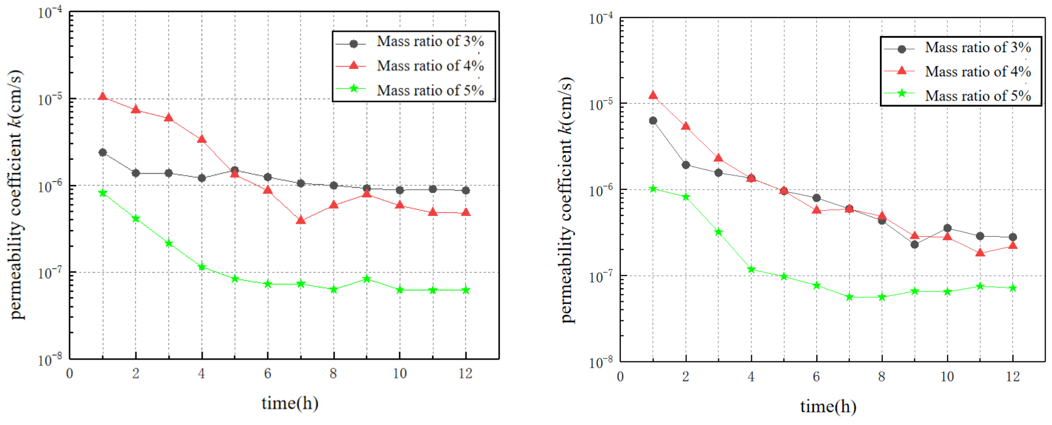

(a) (b)

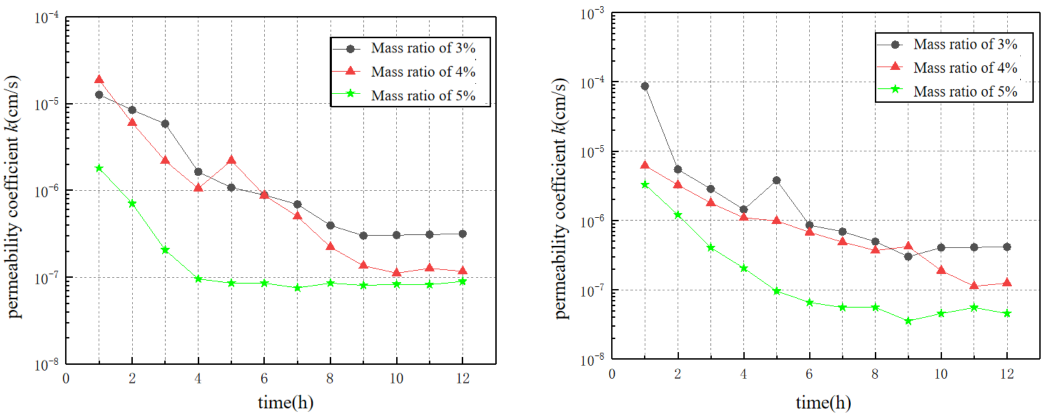

(c) (d)

Fig. S6. Comparison of permeability coefficients for the same pressure difference A, B, C: (a)5kPa; (b)10kPa; (c)15kPa; (d)20kPa.

4. Schematic diagram of marsupial structure

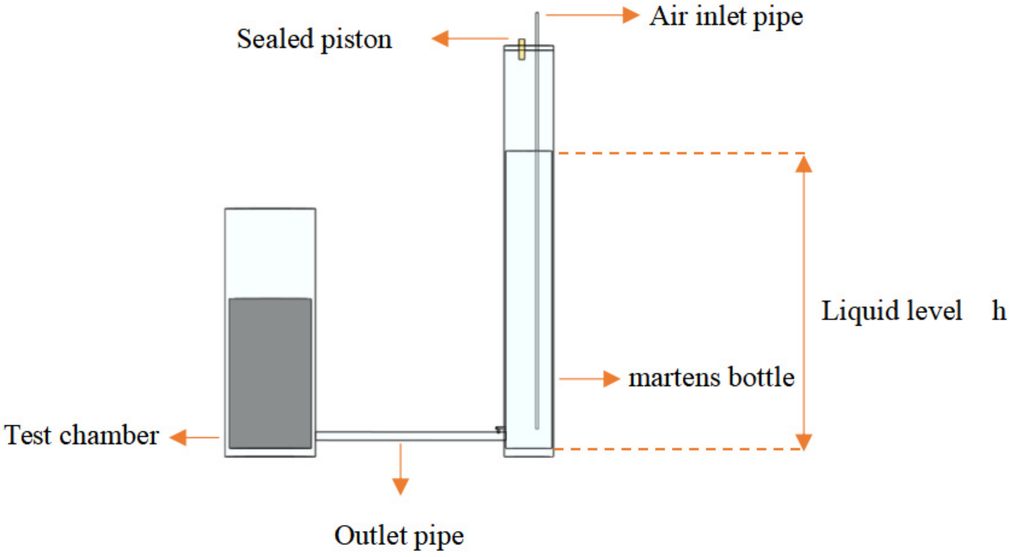

Fig. S7. Schematic diagram of marsupial structure

5. Bayer filters

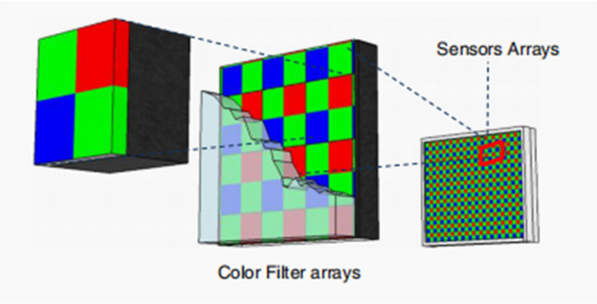

Fig. S8. Common Bayer filters in color cameras

6. Permeation test model diagram

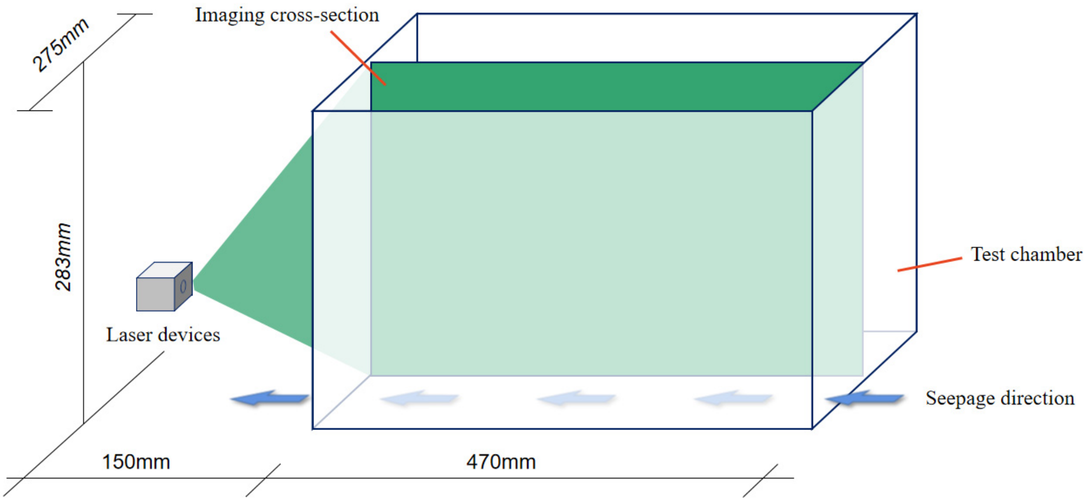

Fig. S9. Schematic diagram of imaging cross-section
